# Computational simulation of pacifier deformation and interaction with the palate

**DOI:** 10.1002/cre2.428

**Published:** 2021-04-06

**Authors:** Christopher L. Lee, Michael Costello, David A. Tesini

**Affiliations:** ^1^ Olin College of Engineering Needham Massachusetts USA; ^2^ Department of Pediatric Dentistry Tufts University School of Dental Medicine Boston Massachusetts USA; ^3^ Toothprints PC Hopkinton Massachusetts USA

**Keywords:** finite element analysis, myofunction, pacifier, palate

## Abstract

**Objectives:**

The objective of this study is to demonstrate that computational finite element models can be used to reliably simulate dynamic interaction between a pacifier, the palate, and the tongue during nonnutritive sucking (NNS). The interactions can be quantified by the results of finite element analyses which include deformation, strain, stress, contact force, and contact area.

**Materials and Methods:**

A finite element model was created based upon CAD solid models of an infant pacifier and palate. The silicone pacifier bulb is represented by a hyperelastic constitutive law. Contact surfaces are defined between the pacifier and palate. A time and spatially varying pressure load is applied to the bulb representing peristaltic interaction with the tongue. A second time‐varying, periodic pressure representing NNS is applied to the model simultaneously. A large displacement, nonlinear transient dynamic analysis is run over two NNS cycles.

**Results:**

Results from the finite element analysis show the deformed shape of the bulb with maximum principal elastic strain of 0.23 and a range of maximum principal stress on the palate from 0.60 MPa (tensile) to −0.27 MPa (compressive) over the NNS cycles. The areas of contact between the pacifier and the palate are shown in surface contour plots.

**Conclusions:**

A nonlinear transient dynamic finite element model can simulate the mechanical behavior of a pacifier and its interaction with the tongue and contact with the palate subject to NNS. Quantitative results predicting deformation, strain, stress, contact force, and contact area can be used in comparative studies to provide insight on how pacifiers cause changes in dental, orthognathic, and facial development.

## 1 INTRODUCTION

This short communication presents a demonstration that computational finite element models can be used to simulate and predict dynamic interaction between a pacifier, the palate, and the tongue during nonnutritive sucking (NNS). This analysis tool enables direct quantification of pacifier deformation and the ensuing contact with the palate. Such knowledge would be useful to guide pacifier design, for comparison to clinical observations and experiments, and to help pediatric dentists and orthodontists understand orthodontic effects of palatal collapse. This common problem with prolonged pacifier use predisposes the child to development of posterior crossbites and other malocclusions (Zardetto et al., 2002).

Finite element analysis (FEA) is used in dentistry primarily in applications related to implants, prothesis, and restorations (Trivedi, 2014). Very few studies have applied FEA to pacifiers. Two that have (Freitas, 2020; Levrini et al., 2007) used a linear elastic material model which is not well‐suited for capturing large strains in silicone rubber (pacifier bulb). In addition, both analyses were static and did not describe dynamics mechanical behavior during a NNS cycle. The primary results of Levrini et al. (2007) are calculations of stress distributions on the palate surface for three different‐shaped pacifiers. Results were presented as contour surface plots. Specific stress values were not reported. The primary results of Freitas (2020) are contour plots of displacement of the pacifier and distributions of force and contact area on the maxillary bone and teeth due to a static force applied from the tongue.

**FIGURE 1:**
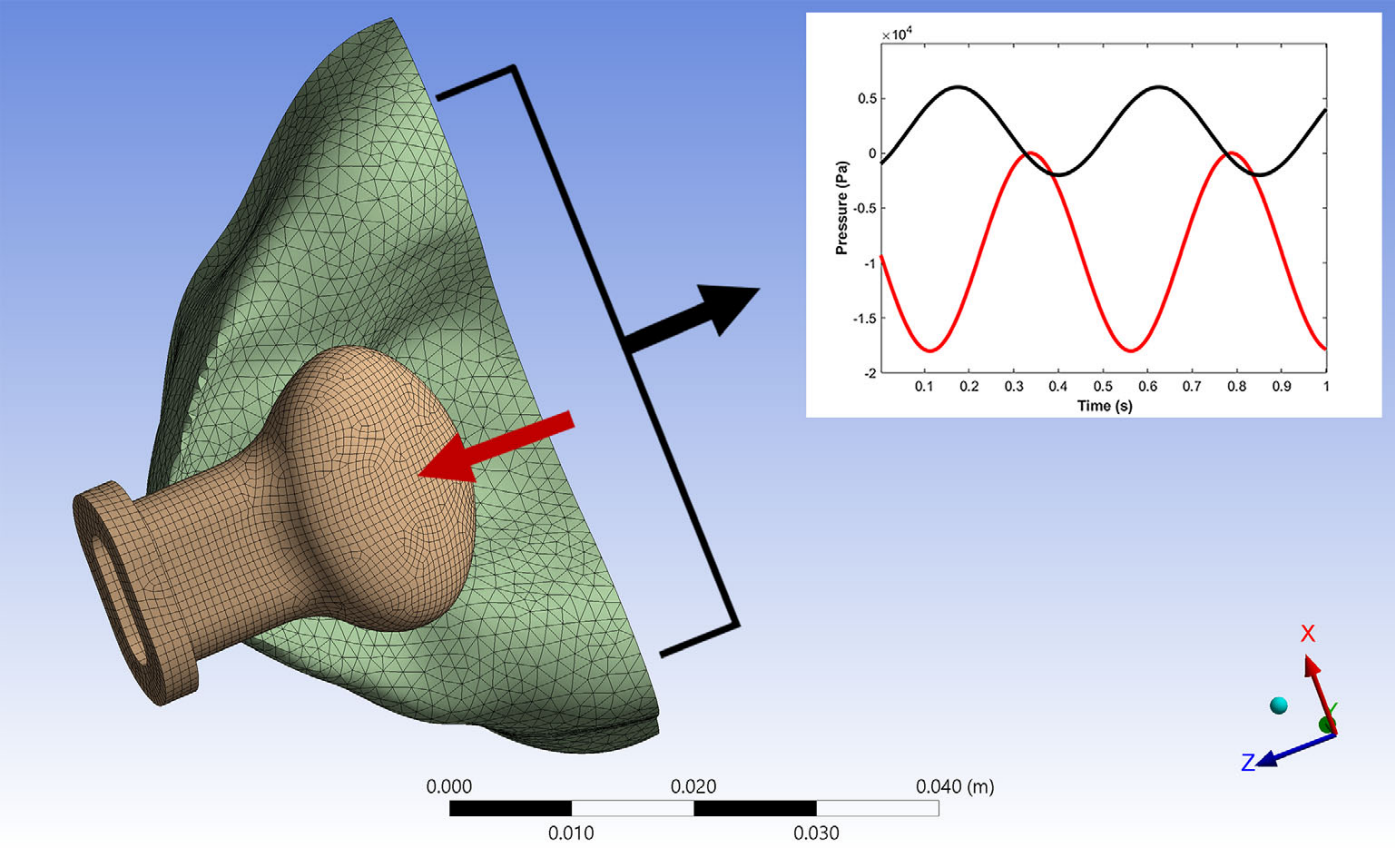
Finite element model with mesh of the pacifier bulb and palate (view of transverse plane at small inclination). Peristaltic pressure (red) is applied on the inferior surface of the bulb. Negative suction pressure (black) is applied to bulb and palate surfaces

The finite element model of this study (see Figure 1) incorporates greater physical detail resulting in a more realistic representation of pacifier–palate mechanics during NNS. The silicone pacifier bulb is represented by as a hyperelastic material which can capture nonlinear elastic behavior. A transient dynamics analysis is run over two NNS cycles. The interaction of the tongue is included as a spatially distributed, time‐varying pressure applied on the inferior side of the pacifier bulb. A second time‐varying pressure is superimposed on the palate and the bulb. These loads represent peristaltic action of the tongue and the negative pressure of the intraoral environment during NNS.

## 2 MATERIALS AND METHODS

The finite element model has two separate parts, the pacifier bulb and the palate. The bulb model was generated from a solid model of an original Closer to Nature soother, size 3 (tommee tippee brand, Mayborn USA Inc., Stamford, CT) and the palate model was based upon a dental impression (3D Scan Services LLC, Mission Viejo, CA). The silicone rubber bulb (density, *ρ* = 1.2 g/cm^3^) is represented using a hyperelastic, five‐parameter Mooney–Rivlin material model with parameter values C10 = 4.49e5 Pa, C01 = −1.03e5 Pa, C20 = 3.16e4 Pa, C11 = 1.37e3 Pa, and C02 = −0.33 Pa. These parameters were determined by curve fitting uniaxial tension and biaxial tension test data from silicone rubber samples. Volumetric test data was not available so the incompressibility parameter D, was taken to be zero. The palate is represented as a linear elastic material with property values from metrology measurements from palatine process of maxilla (*ρ* = 2000 kg/m^3^, *E* = 6e9 Pa, *ν* = 0.45, *β* = 2e10 Pa) (Chen et al., 2015; Peterson et al., 2005). The finite element mesh (shown in Figure 1) consists of 117,639 nodes and 59,018 hex‐dominated elements.

The bulb is constrained (fixed—no translation or rotation) at its base and the palate is fixed on the superior horizontal surface. Initially the pacifier bulb is not in contact with the palate. The base surface of the pacifier is placed at a 10° angle with respect to vertical and at a distance of 6.6 mm from the anterior edge of the palate. The contact pressure from the tongue and the intraoral sucking pressure are sinusoidal and are applied on the inferior side of the bulb (4 kPa magnitude and 0.73 s period) and on the palate and bulb (9 kPa magnitude and 0.675 s period), respectively. This loading is based on measurements reported in (Capilouto et al., 2014; Chen et al., 2018; Lindner & Hellsing, 1991; Nishi et al., 2016). The superior surface of the bulb and the palate surface are defined as frictionless contact surfaces. Additionally, the inside wall of the pacifier bulb is defined as a single contact surface. Under large pressure on its outside surface, the bulb can collapse allowing its interior to come in contact with itself. Nonlinear, large displacement, transient dynamic analyses were performed using ANSYS Workbench (ver. 18.2) for two NNS cycles.

## 3 RESULTS

**FIGURE 2:**
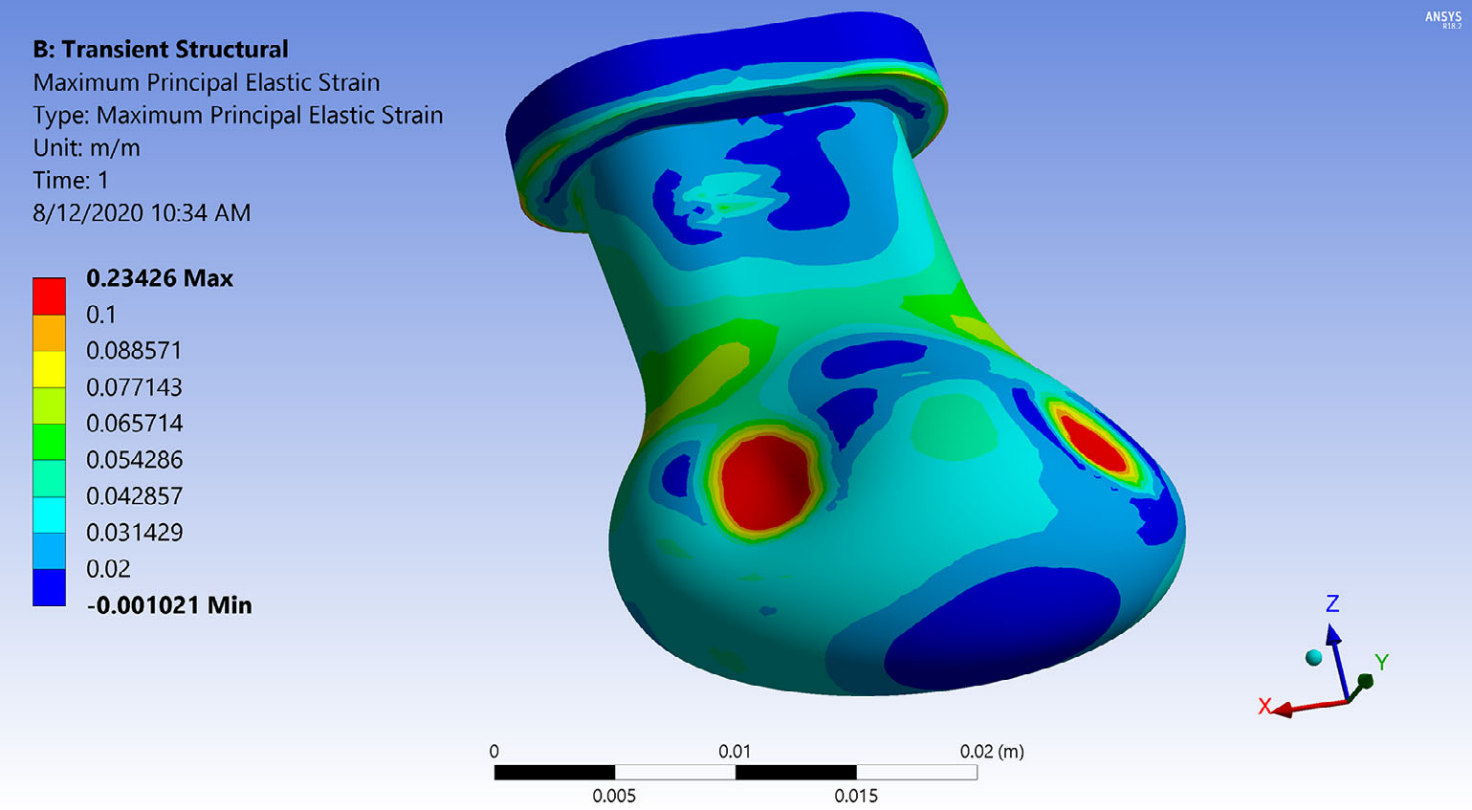
Contours of maximum principal elastic strain on the deformed shape of the bulb

**FIGURE 3:**
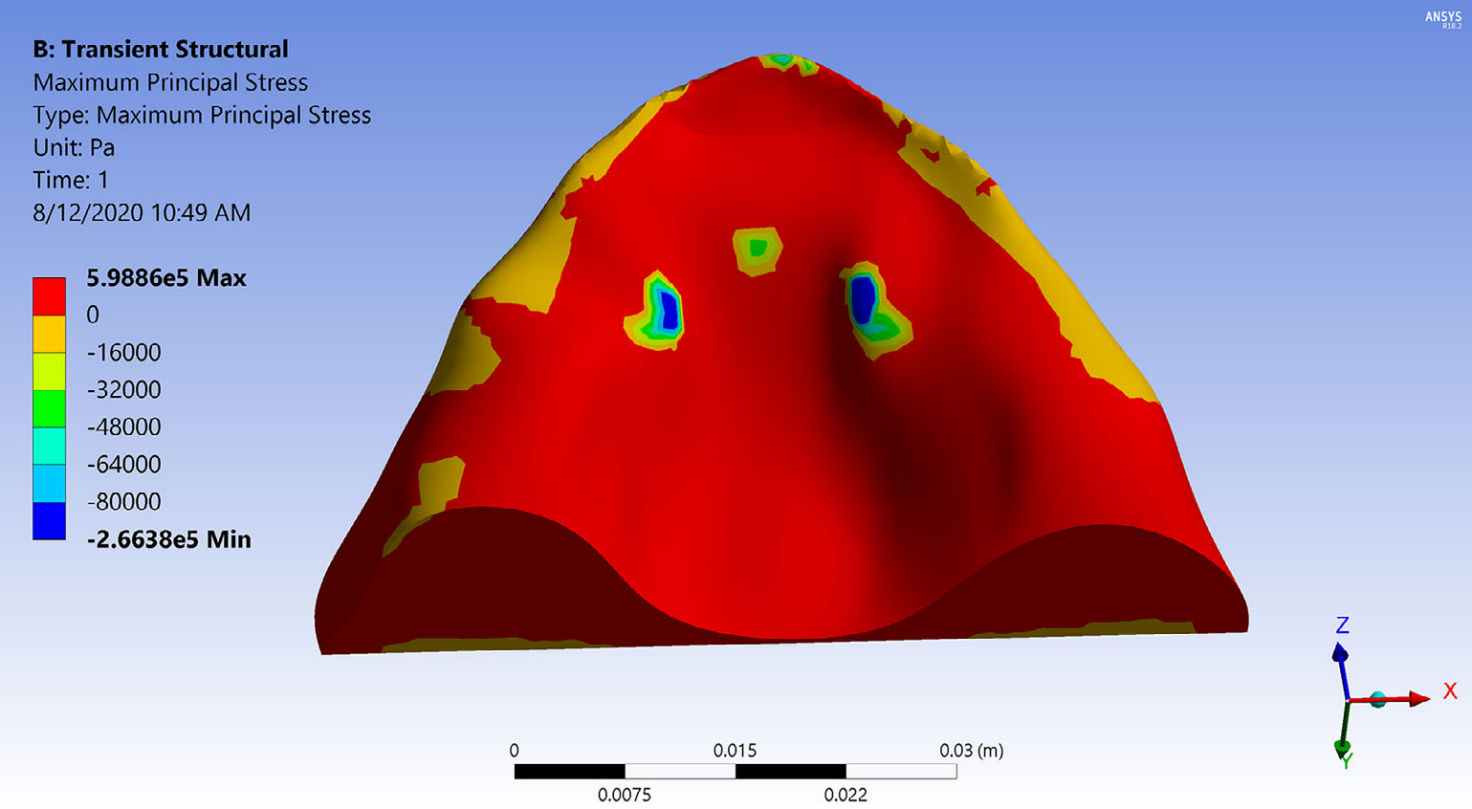
Contours of maximum principal stress on the palate caused by contact from the pacifier

**FIGURE 4:**
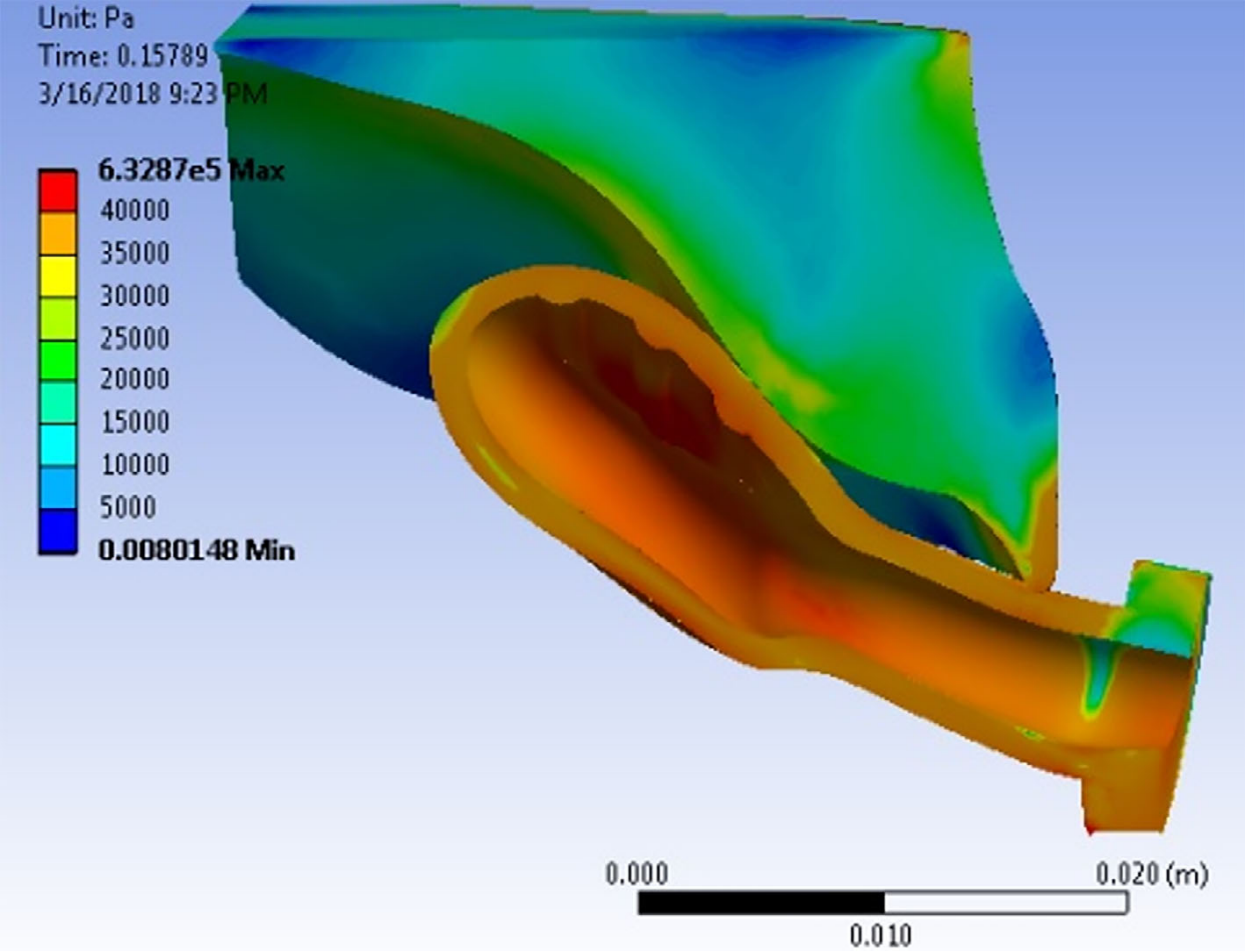
Section view cut by the median plane. Stress contours superimposed on the deformed bulb which is in contact with the palate

Simulations show that the tongue pushes the pacifier bulb into contact with the palate after which the bulb deforms due to the contact and applied pressure loading. The results including the total deformation, effective strain, principal stresses, contact pressure, and contact area are calculated at specified time steps during the simulation. Representative strain and stress contours are presented. Figure 2 shows principal elastic strain (maximum value 0.23) of the bulb with color contours superimposed on its deformed shape at the final time step. The highest strain is concentrated in three areas of contact, two on either side of the bulb and one in the middle. Figure 3 shows the corresponding maximum principal stress (0.60 MPa tensile and − 0.27 MPa compressive) on the palate. Figure 4 shows stress contours in a section view cut by the median plane. The deformed pacifier bulb is in contact with the palate.

## 4 DISCUSSION

The results of the sample case presented above demonstrate that finite element simulations can predict the dynamic deformation of a pacifier bulb and palate when subject to pressure loading from peristalsis of the tongue and from intraoral pressure of NNS and calculate accompanying stresses, strains, pressures, and contact areas. Finite element analysis can be used as an investigative tool to evaluate effects changes in size, geometry, relative positions, loads, and materials have on the resulting forces, pressures, and total contact area between the pacifier bulb and the palate, especially in the tektal area of the palate as defined by Hohoff et al. (2005). Computational results can be compared to experimental and clinical studies of the mechanical behavior of pacifiers thereby increasing understanding of how pacifiers cause changes in dental, orthognathic and facial development.

## CONFLICT OF INTEREST

David A. Tesini has a patent US Patent No. 7,731,773 with royalties paid to Smilo, a patent US Patent No. 7,931,672 with royalties paid to Smilo, a patent US Patent No. 7,883,530 with royalties paid to Smilo, and a patent US Patent No. 9,515,815 with royalties paid to Tomy.

## AUTHOR CONTRIBUTIONS

C.L. wrote, edited, and revised the manuscript; and analyzed and evaluated the results. M.C. set‐up and conducted the computational simulations. D.T. conceived the idea for the study; and wrote, edited, and revised the manuscript. All authors approved the final version of the manuscript.

## Data Availability

The data that support the findings of this study are available from the corresponding author upon reasonable request.

## References

[cre2428-bib-0001] Capilouto, G. J. , Cunningham, T. , Frederick, E. , Dupont‐Versteegden, E. , Desai, N. , & Butterfield, T. A. (2014). Comparison of tongue muscle characteristics of preterm and full term infants during nutritive and nonnutritive sucking. Infant Behavior and Development, 37, 435–445. 24956503 10.1016/j.infbeh.2014.05.010

[cre2428-bib-0002] Chen, J. , Ahmad, R. , Li, W. , Swain, M. , & Li, Q. (2015). Biomechanics of oral mucosa. Journal of the Royal Society Interface, 12, 109. 10.1098/rsif.2015.0325 PMC4535403 26224566

[cre2428-bib-0003] Chen, L. , Lucas, R. F. , & Feng, B. (2018). A novel system to measure infants' nutritive sucking during breastfeeding: The breastfeeding diagnostic device (BDD). IEEE Journal of Translational Engineering in Health and Medicine, 6, 2700208. 29888144 10.1109/JTEHM.2018.2838139 PMC5991864

[cre2428-bib-0004] Freitas, C. (2020). Avaliaçăo da Rugosidade Superficial e da Distribuiçăo de Tensoes Mecănicas Sobre o Palato de Diferentes Tipos De Chupetas [Assessment of surface roughness and the distribution of mechanical stress on the palate by different pacifiers] (doctorate in dentistry thesis). Universidade Estadual de Campinas. Retrieved from http://repositorio.unicamp.br

[cre2428-bib-0005] Hohoff, A. , Rabe, H. , Ehmer, U. , & Harms, E. (2005). Palatal development of preterm and low birthweight infants compared to term infants – What do we know? Part 1: The palate of the term newborn. Head & Face Medicine, 1(1), 1–11. 16270908 10.1186/1746-160X-1-8 PMC1308841

[cre2428-bib-0006] Levrini, L. , Merlo, L. , & Paracchini, L. (2007). Different geometric patterns of pacifiers compared on the basis of finite element analysis. European Journal of Paediatric Dentistry, 4, 173–178. 18163851

[cre2428-bib-0007] Lindner, A. , & Hellsing, E. (1991). Cheek and lip pressure against maxillary dental arch during dummy sucking. European Journal of Orthodontics, 13(5), 362–366. 1748182 10.1093/ejo/13.5.362

[cre2428-bib-0008] Nishi, E. , Nagamatsu, Y. , & Nikawa, T. (2016). Measurement of force applied by infant tongue to the nipple during sucking and investigation of the mechanism of tongue movement. In Conference proceedings of the 38th annual international conference of the IEEE engineering in medicine and biology society, August 16–20. IEEE. 10.1109/EMBC.2016.7591128 28268732

[cre2428-bib-0009] Peterson, J. , Wang, Q. , & Dechow, P. C. (2005). Material properties of the dentate maxilla. The Anatomical Record, 288A(9), 962–972. 10.1002/ar.a.20358 16894571

[cre2428-bib-0010] Trivedi, S. (2014). Finite element analysis: A boon to dentistry. Journal of Oral Biology and Craniofacial Research, 4, 200–203. 25737944 10.1016/j.jobcr.2014.11.008 PMC4306993

[cre2428-bib-0011] Zardetto, C. G. , Rodrigues, C. R. , & Stefani, F. M. (2002). Effects of different pacifiers on the primary definition and oral myofunctional structures of preschool children. Pediatric Dentistry, 24(6), 552–560. 12528948

